# Follow-up plasma apolipoprotein E levels in the Australian Imaging, Biomarkers and Lifestyle Flagship Study of Ageing (AIBL) cohort

**DOI:** 10.1186/s13195-015-0105-6

**Published:** 2015-02-20

**Authors:** Veer B Gupta, Andrea C Wilson, Samantha Burnham, Eugene Hone, Steve Pedrini, Simon M Laws, Wei Ling Florence Lim, Alan Rembach, Stephanie Rainey-Smith, David Ames, Lynne Cobiac, S Lance Macaulay, Colin L Masters, Christopher C Rowe, Ashley I Bush, Ralph N Martins, for the AIBL Research Group

**Affiliations:** Centre of Excellence in Alzheimer’s Disease Research and Care, School of Medical Sciences, Edith Cowan University, 270 Joondalup Drive, Joondalup, 6027 Australia; McCusker Alzheimer’s Research Foundation, Hollywood Medical Centre, 85 Monash Avenue, Suite 22, Nedlands, 6009 Australia; CSIRO Computational Informatics, Preventative Health Flagship, 65 Brockway Road, Floreat, 6014 Australia; Cooperative Research Centre for Mental Health, Carlton, VIC 3053 Australia; Florey Institute of Neuroscience and Mental Health, Parkville, VIC 3052 Australia; Academic Unit for Psychiatry of Old Age, Department of Psychiatry, The University of Melbourne, St Vincent’s Aged Psychiatry Service, St George’s Hospital, Melbourne, VIC 3065 Australia; National Ageing Research Institute, Parkville, VIC 3052 Australia; CSIRO Preventative Health Flagship, Adelaide, SA 5000 Australia; CSIRO, Parkville, VIC 3052 Australia; Mental Health Research Institute, The University of Melbourne, Parkville, VIC 3052 Australia; Centre for Neuroscience, The University of Melbourne, Parkville, VIC 3010 Australia; Department of Nuclear Medicine & Centre for PET, Austin Health, Heidelberg, VIC 3084 Australia; School of Psychiatry and Clinical Neurosciences, The University of Western Australia, Nedlands, 6009 Australia

## Abstract

**Introduction:**

Alzheimer’s disease (AD) is a growing socioeconomic problem worldwide. Early diagnosis and prevention of this devastating disease have become a research priority. Consequently, the identification of clinically significant and sensitive blood biomarkers for its early detection is very important. Apolipoprotein E (*APOE*) is a well-known and established genetic risk factor for late-onset AD; however, the impact of the protein level on AD risk is unclear. We assessed the utility of plasma ApoE protein as a potential biomarker of AD in the large, well-characterised Australian Imaging, Biomarkers and Lifestyle Study of Ageing (AIBL) cohort.

**Methods:**

Total plasma ApoE levels were measured at 18-month follow-up using a commercial bead-based enzyme-linked immunosorbent assay: the Luminex xMAP human apolipoprotein kit. ApoE levels were then analysed between clinical classifications (healthy controls, mild cognitive impairment (MCI) and AD) and correlated with the data available from the AIBL cohort, including but not limited to *APOE* genotype and cerebral amyloid burden.

**Results:**

A significant decrease in ApoE levels was found in the AD group compared with the healthy controls. These results validate previously published ApoE protein levels at baseline obtained using different methodology. ApoE protein levels were also significantly affected, depending on *APOE* genotypes, with ε2/ε2 having the highest protein levels and ε4/ε4 having the lowest. Plasma ApoE levels were significantly negatively correlated with cerebral amyloid burden as measured by neuroimaging.

**Conclusions:**

ApoE is decreased in individuals with AD compared with healthy controls at 18-month follow-up, and this trend is consistent with our results published at baseline. The influence of *APOE* genotype and sex on the protein levels are also explored. It is clear that ApoE is a strong player in the aetiology of this disease at both the protein and genetic levels.

## Introduction

Current research in the area of Alzheimer’s disease (AD) indicates an urgent need for the discovery and validation of sensitive and specific protein biomarkers for the early detection and treatment of this devastating disease [1,2]. Pathological changes in brain amyloid-β (Aβ) protein deposits visualised by neuroimaging are captured up to 15 years prior to the manifestation of clinical symptoms [3-5]. With definitive diagnosis possible only post-mortem, it is critical that more inexpensive and widely accessible strategies be developed to capture these changes early. Blood-based biomarkers are currently being mined for utility in detecting preclinical AD, where the goal is to develop a screening tool in the form of a routine blood test for early diagnosis.

Apolipoprotein E (ApoE) is a well-defined genetic risk factor for late-onset AD [6]. The human *APOE* gene has three polymorphic alleles—namely ε2, ε3 and ε4 [7]—where an individual acquires two alleles resulting in six different phenotypes: ε2/ε2, ε2/ε3, ε3/ε3, ε2/ε4, ε3/ε4 and ε4/ε4. Importantly, approximately 50% of AD patients carry the ε4 allele (compared with 14% in the general population), with the majority being heterozygotes (ε3/ε4 [8,9]). The *APOE*-ε4 allele has been implicated in many AD pathological pathways. Furthermore, the number of inherited ε4 alleles is associated with both increased disease risk and decreased average age of onset compared with inheritance of the ε2 or ε3 alleles [8].

Biologically, the ApoE protein is known to influence lipid homeostasis by regulating lipid transport, such as cholesterol, in an isoform-dependent manner [10-12]. The differences between the three ApoE isoforms are based on two amino acids that affect its structure and hence the interaction and binding of the protein with various lipids and Aβ [13-15]. Histologically, ApoE and Aβ can co-localise in the brain, and therefore their complementary roles have been studied extensively [16,17]. These studies have led to the association of the ApoE4 protein with lower Aβ_1–42_ and higher tau levels observed in cerebrospinal fluid, increased brain atrophy and increased neocortical amyloid burden [7,18-20]. In contrast, ApoE2 is considered to be more cognitively protective than ApoE4; however, this is seemingly independent of actual Aβ pathology in the brain [13,21].

Whilst the *APOE* gene is considered one of the strongest risk factors for late-onset AD, the mechanisms and influence of actual plasma ApoE levels on the pathophysiology of AD remain unclear and require further elucidation. Therefore, we measured ApoE protein levels in plasma to further assess and determine the diagnostic value of ApoE as an AD blood biomarker. Given the prior knowledge of ApoE involvement in Aβ metabolism mentioned above, we also evaluated the association of plasma ApoE on neocortical Aβ burden as measured by positron emission tomography (PET).

Previously, we reported on the baseline ApoE data derived from the Australian Imaging, Biomarkers and Lifestyle Study of Ageing (AIBL) and showed a significant decrease in ApoE protein levels in the AD group compared with the cognitively ‘normal’ controls [22]. In the present study, we quantified ApoE data in the same subjects after 18 months of follow-up by utilising a more advanced enzyme-linked immunosorbent assay technology. This study not only validates our data in a time-dependent manner, within the same cohort, but also illustrates reproducibility with a different experimental method.

## Methods

### The AIBL cohort

The cohort recruitment process, including neuropsychological, lifestyle and mood assessments, have been described in detail previously [23]. In brief, in the AIBL study, researchers recruited a total of 1,166 participants over the age of 60 years at baseline, of whom 54 were excluded because of comorbid disorders or consent withdrawal. Using the National Institute of Neurological and Communicative Disorders and Stroke/Alzheimer’s Disease and Related Disorders Association international criteria for AD diagnosis [24], a clinical review panel determined disease classifications at each assessment time point to ensure accurate and consistent diagnoses amongst the participants. According to these diagnostic criteria, participants were classified into one of three groups; AD, mild cognitive impairment (MCI) or healthy controls (HC). At baseline, there were a total of 768 HC, 133 subjects with MCI and 211 subjects with AD.

The AIBL study is a prospective, longitudinal study, following participants at 18-month intervals. In this report, we describe findings for 954 individuals who completed the full study assessment and corresponding blood sample collection at both baseline and 18-month follow-up. Of these 954 participants, 689 were classified as HC, 78 as MCI and 187 as AD.

The institutional ethics committees of Austin Health, St. Vincent’s Health, Hollywood Private Hospital and Edith Cowan University granted ethical approval for the AIBL study. All volunteers gave their written informed consent prior to participating in the study.

### Sample collection and *APOE* genotyping

Plasma was isolated from whole blood and collected in standard ethylenediaminetetraacetic acid tubes with prostaglandin E_1_ (33.3 ng/ml; Sapphire Biosciences, Waterloo, Australia) added. Upon completion of blood fractionation, samples were aliquoted and immediately stored in liquid nitrogen until required for analysis. DNA was isolated from whole blood using a QIAamp DNA Blood Midi Kit (Qiagen, Chadstone Centre, Australia) according to the manufacturer’s protocol, and *APOE* genotype was determined through either PCR amplification and restriction enzyme digestions, as previously described [25], or through TaqMan genotyping assays (Life Technologies, Mulgrave, Australia) for rs7412 (Assay ID: C____904973_10) and rs429358 (Assay ID: C_3084793_20). For TaqMan assays, PCRs and real-time fluorescence measurements were carried out on a ViiA 7 real-time PCR system (Applied Biosystems, Mulgrave, Australia) using the TaqMan GTXpress Master Mix (Life Technologies) methodology per the manufacturer’s instructions.

### Total apolipoprotein E assay

Total plasma ApoE levels were measured using a commercial Luminex xMAP Human apolipoprotein kit (EMD Millipore, Billerica, MA, USA), a bead-based assay. This kit uses capture antibodies on the surface of fluorescently coated beads. Each microsphere is conjugated with a specific capture antibody—in this case, a specific human anti-ApoE antibody. Briefly, the plasma samples were thawed on ice, centrifuged for 10 minutes at 12,000 × *g* and diluted 10,000-fold using the supplied assay buffer diluents. Quality control and human ApoE calibrators were reconstituted in deionized water to give working solutions. Antibody-immobilised beads were prepared separately. The beads were vortexed for 1 minute and then incubated in a sonicating bath for 8 to 10 minutes. The beads were diluted using the provided diluents, and the final solution was sonicated and vortexed again just prior to loading onto the plate. Finally, all reagents were loaded onto the provided filter plate in the appropriate proportions as per the kit instructions, incubated for 1 hour and then vacuum-drained and washed. The detection antibodies and streptavidin-phycoerythrin were added for 30 minutes and vacuumed and washed in the same manner. Plates were read on the Bio-Plex 200 multiplexing instrument (Bio-Rad Laboratories, Gladesville, Australia). The assay sensitivity for ApoE was 0.10 ng/ml, and the intra-assay and inter-assay precisions were 5% and 22%, respectively.

### Brain imaging in a subset of the AIBL cohort

A subset of the AIBL cohort (n = 287) underwent carbon-11-labeled Pittsburgh Compound B positron emission tomography (^11^C-PiB-PET) imaging at baseline to measure cerebral amyloid load as previously described [26]. PET standardised uptake value (SUV) data were summed and normalised to the cerebellar cortex SUV to form the region to cerebellar ratio (SUVR). Of the total 954 participants reported on here, 217 underwent PiB-PET imaging at 18-month follow-up.

### Statistical analysis

Differences in demographics across clinical categories were assessed using one-way analysis of variance (ANOVA) for continuous data (age) and *χ*^2^ tests for categorised data (sex and *APOE*-ε4 carriage). Differences in ApoE levels between clinical classifications were assessed using ANOVA. Tukey’s honestly significant difference (HSD) *post hoc* adjustment was applied to individual classification differences.

General linear models were used to assess correlations between SUVR and ApoE levels, and correlation coefficients (β) and ApoE level specific *P*-values are reported. Receiver operating characteristic curves for predicting PiB status were calculated from predictions given by tenfold cross-validated random forest models, which have been shown previously [27] to have efficacy in creating blood-based predictors for PiB status. All statistical analyses were conducted using R software version 2.15.1 [28].

## Results

Demographic data for the 18-month follow-up, including number of participants (both female and male) in each of the clinical categories, *APOE*-ε4 status, mean age and AIBL cohort are presented in Table 1. The average age was significantly higher in individuals with MCI (77.97 years) and AD (80.32 years) than in the HC category (73.51 years) (*P* < 0.001, one-way ANOVA). The percentage of *APOE*-ε4-positive individuals was significantly higher in the AD (68.4%) and MCI (41%) groups than in the HC group (26.9%). The number of female participants was generally higher than males in each of the clinical classification categories.

**Table 1:** **Demographic characteristics, including** ***APOE*** **-ε4 frequency, of the study groups** ^**a**^

**Categories**	**HC**	**MCI**	**AD**	***P*** **-value**
Count, *n*	689	78	187	
Age, yr	73.51 ± 6.78	77.97 ± 7.58	80.32 ± 7.79	<0.001 (one-way ANOVA)
Sex, M/F	285/404	37/41	76/111	0.556 (*χ* ^2^ test)
*APOE*-ε4-positive, %	26.9	41	68.4	<0.001 (*χ* ^2^ test)

^a^AD, Alzheimer’s disease; APOE, Apolipoprotein E; HC, Healthy controls; MCI, Mild cognitive impairment. Values are mean ± standard deviation or ratio (%). Statistical analysis of age (in years) and sex of the participants was carried out using one-way analysis of variance (ANOVA), and *APOE*-ε4 genotype frequency was performed using the *χ* ^2^ test.

**Figure 1:**
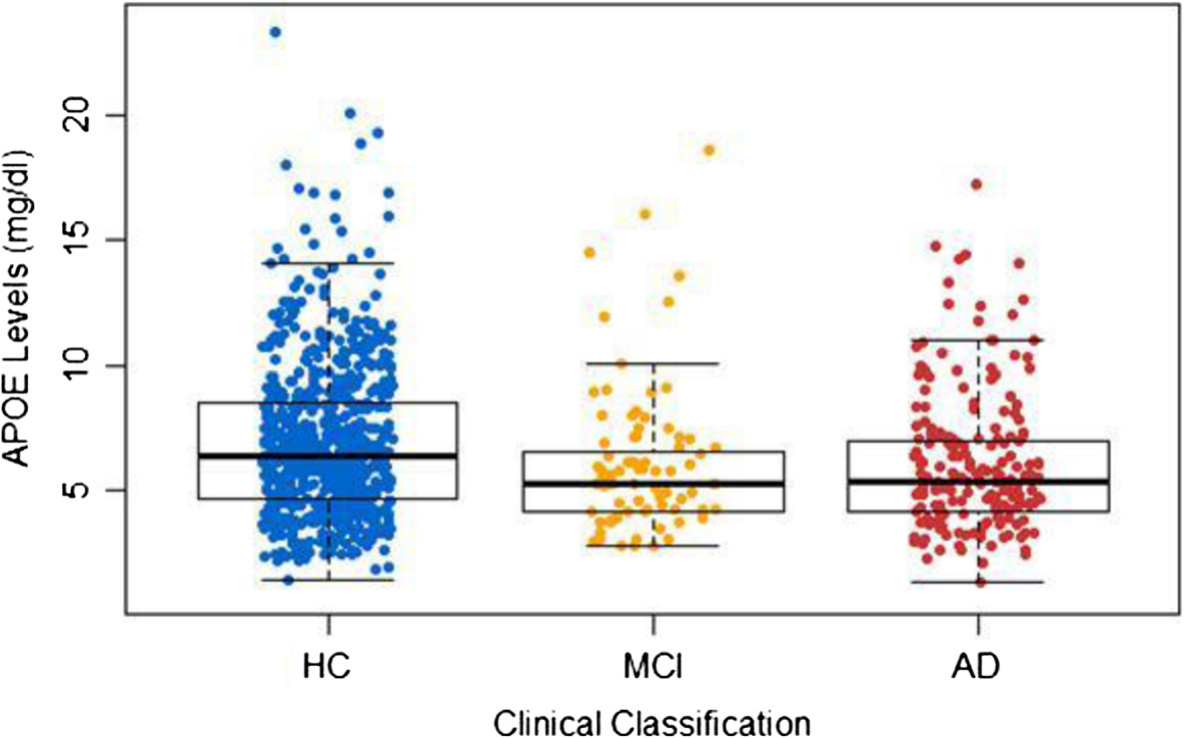
**Apolipoprotein E protein levels across all clinical classifications in the AIBL cohort at 18 months.** Data are presented as mean ± standard deviation for absolute apolipoprotein E (ApoE) levels (mg/dl) across clinical classification categories of the Australian Imaging, Biomarkers and Lifestyle Study of Ageing (AIBL) cohort participants at the 18-month time point. One-way analysis of variance overall *P* = 0.002, overall adjusted *P* = 0.001 (adjusted for age, sex and *APOE*-ε4 genotype), followed by *post hoc* Tukey’s honestly significant difference test: healthy controls (HC) versus Alzheimer’s disease (AD), *P* = 0.005; HC versus mild cognitive impairment (MCI), *P* = 0.997; and MCI versus AD, *P* = 0.064.

**Figure 2:**
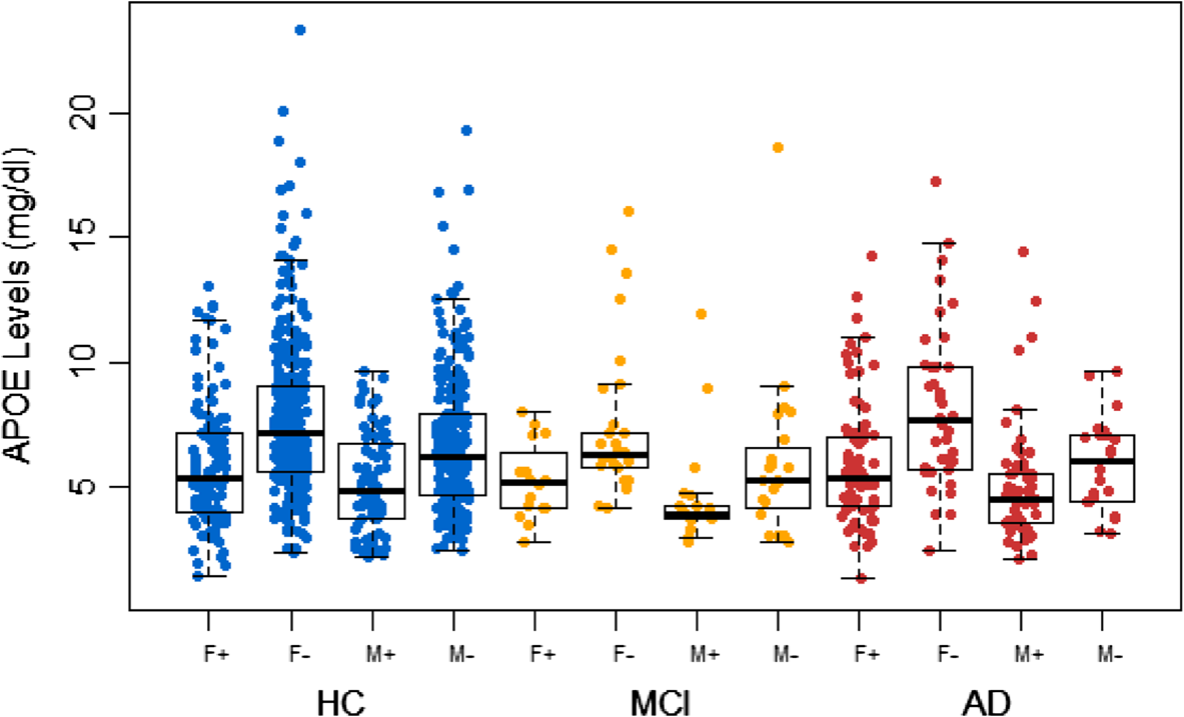
**Absolute apolipoprotein E levels across clinical categories.** Data are presented as means ± standard deviations of apolipoprotein (ApoE) levels stratified by sex and *APOE*-ε4 genotype in the Australian Imaging, Biomarkers and Lifestyle Study of Ageing (AIBL) cohort participants at the 18-month time point. One-way analysis of variance followed by *post hoc* Tukey’s honestly significant difference test was carried out. Healthy controls (HC): females, *P* < 0.001 for ε4 (F+) versus non-ε4 (F−); males, *P* < 0.001 for ε4 (M+) versus non-ε4 (M−). Mild cognitive impairment (MCI): females, *P* = 0.040 for ε4 versus non-ε4; males, *P* = 0.539 for ε4 versus non-ε4. Alzheimer’s disease (AD): females, *P* < 0.001 for ε4 versus non-ε4; males, *P* = 0.604 for ε4 versus non-ε4.

**Figure 3:**
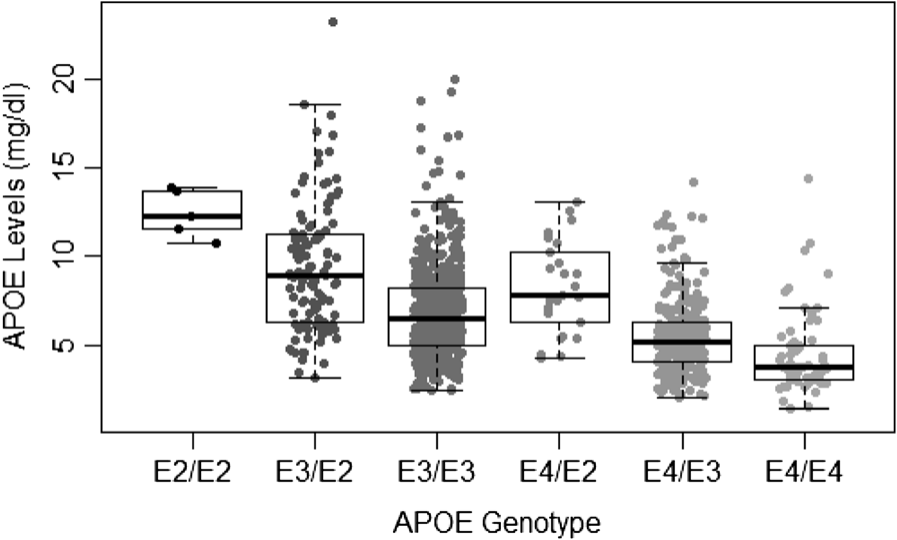
**Apolipoprotein E protein levels across all individual** ***APOE*** **genotypes.** Data are presented as mean ± SD for plasma apolipoprotein E (ApoE) protein levels (mg/dl) across all *APOE* genotype combination categories in the Australian Imaging, Biomarkers and Lifestyle Study of Ageing (AIBL) cohort participants at the 18-month time point. One-way analysis of variance overall *P* < 0.001, overall adjusted *P* < 0.001 (adjusted for age, sex and clinical classification). *Post hoc* Tukey’s honestly significant difference test results between genotypes are shown in Table 3.

ApoE levels were significantly different across the diagnostic classification categories (*P* = 0.002) (Figure 1). When controlling for age, sex and *APOE*-ε4 status, ApoE levels remained significantly different between clinical classifications (p < 0.001). Post-hoc analysis (Tukey’s HSD) revealed that lower ApoE levels were seen in individuals with AD (6.20 mg/dl) when compared with HC (6.97 mg/dl; p = 0.005). Total ApoE levels were significantly decreased in *APOE*-ε4 carriers (5.59 mg/dl) compared with non-carriers (7.41 mg/dl; p < 0.001; Table 2) and remained so after correction for known pre-disposing factors, age and sex. This relationship also remained strong when the participants were stratified by clinical classification (with or without correction for age and sex) as well as stratification by sex (with or without correction for age); refer to Table 2 and Figure 2. Further, males had lower total ApoE levels (6.12 mg/dl) compared with females (7.21 mg/dl; p = 0.013) irrespective of clinical classification. ApoE levels were seen to have significant (p < 0.05) differences across ApoE genotype classifications by one-way ANOVA with Tukey’s HSD post-hoc adjustment, refer to Figure 3 and Table 3. The only exceptions were between ε2/ε2 and ε2/ε3; ε3/ε4 and ε4/ε4 as well as comparisons with ε2/ε4.

**Table 2:** **Comparison of apolipoprotein E levels among different clinical classification and sex categories**

**Categories**	**Total**	**Non-ε4 carriers**	**ε4 carriers**	***P*** **crude** ^**a**^	***P*** **adj** ^**a**^
Total ApoE levels (mg/dl)	6.75 ± 3.00	7.41 ± 3.11	5.59 ± 2.40	<0.001	<0.001^b^
Total ApoE levels (HC)	6.97 ± 3.02	7.45 ± 3.10	5.65 ± 2.37	<0.001	<0.001^b^
Total ApoE levels (MCI)	6.17 ± 3.02	6.96 ± 3.37	5.02 ± 1.97	0.005	0.005^b^
Total ApoE levels (AD)	6.20 ± 2.81	7.39 ± 3.05	5.65 ± 2.53	<0.001	<0.001^b^
Total ApoE levels (female)	7.21 ± 3.10	7.91 ± 3.18	5.94 ± 2.87	<0.001	<0.001^c^
Total ApoE levels (male)	6.12 ± 2.74	6.70 ± 2.49	5.12 ± 2.18	<0.001	<0.001^c^

AD, Alzheimer’s disease; HC, Healthy controls; MCI, Mild cognitive impairment, ^a^Comparison between apolipoprotein E (*APOE*)-ε4 carriers and non-ε4 carriers. ^b^ *P*-values adjusted after controlling for age and sex. ^c^ *P*-values adjusted after controlling for age.

**Table 3:** **Comparison of apolipoprotein E levels among different** ***APOE*** **genotype categories**

	**ε2/ε2**	**ε2/ε3**	**ε3/ε3**	**ε2/ε4**	**ε3/ε4**
Count, n	5	113	491	25	266
ε2/ε3	0.118				
ε3/ε3	<0.001	<0.001			
ε2/ε4	0.020	0.474	0.156		
ε3/ε4	<0.001	<0.001	<0.001	<0.001	
ε4/ε4, N = 55	<0.001	<0.001	<0.001	<0.001	0.261

*P*-values shown were calculated using one-way analysis of variance (ANOVA) followed by Tukey’s honestly significant difference *post hoc* test. Refer to Figure 3.

**Figure 4:**
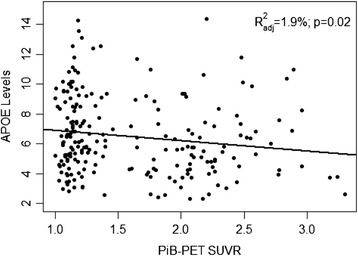
**Correlation between total plasma apolipoprotein E levels (mg/dl) and cerebral amyloid burden as measured by carbon-11-labeled Pittsburgh Compound B positron emission tomography (PiB-PET).** APOE, Apolipoprotein E; SUVR, Standardised uptake value ratio. *R* ^2^_adj_ = 1.9%, *P* = 0.02.

There was a significant association between ApoE and SUVR, with an increase in ApoE levels being associated with a decrease in SUVR (β = −0.034, *P* = 0.025) (Figure 4). Within different clinical categories, however, no correlation was observed between the two groups. The relationship was still evident when we corrected for age and sex in the model (β = −0.040, *P* = 0.009).

When ApoE levels were used to predict PiB-positive and PiB-negative status based on a cutoff of 1.5 SUVR [3], the area under the curve (AUC) was 59.83% (95% confidence interval (CI): 51.89% to 67.77%), with a sensitivity of 57.68% (95% CI: 51.76% to 63.60%) and a specificity 56.76% (95% CI: 50.61% to 62.91%). When *APOE* genotype, age, sex and site were also added to the prediction model, the sensitivity and specificity were increased to 67.77% (95% CI: 66.29% to 69.25%) and 67.04% (95% CI: 65.57% to 68.53%), respectively, with an AUC of 79.12% (95% CI: 78.14% to 80.10%). With regard to neocortical burden, a model for *APOE* genotype, age, sex and site alone obtained sensitivity of 69.74% (95% CI: 63.27% to 76.21%) and specificity of 67.76% (95% CI: 62.34% to 73.18%), respectively, with an AUC of 76.29% (95% CI: 69.54% to 83.05%).

## Discussion

In this study, we expanded the analysis of ApoE in a very well-characterised and well-described cohort, AIBL [23]. Plasma ApoE levels were measured at the 18-month time point. The multitude of data available for each of the AIBL participants allowed us to explore the relationship between ApoE protein levels and other related phenotypes to further reveal the pathways responsible for the onset of AD. Whilst *APOE* genotype plays a significant role in determining the risk of an individual developing AD, the role of ApoE at the protein level is not yet fully understood, and reported results have varied in the literature thus far.

The present study completely corroborates our previous findings that ApoE levels are significantly decreased in the MCI and AD groups compared with the HC [22]. These results remained significant even when we controlled for age, sex and *APOE*-ε4 status. As the AIBL study is a longitudinal prospective study, these 18-month data validate the published baseline data for the same participants and are consistent with other studies [22,29-31]. Further, this report illustrates that ApoE levels are significantly decreased in *APOE*-ε4 carriers, even when stratified by clinical classification or sex. This was also consistent with our previously published data on baseline plasma ApoE [22]. These significant differences observed between the sexes are also supported by recent publications [31,32] in which authors have suggested that the *APOE*-ε4 link to AD is stronger in women. Those who are *APOE*-ε4 carriers have significantly lower ApoE central nervous system (CNS) and plasma ApoE levels, which may explain their propensity to develop AD [22,33,34]. In this study, ApoE protein plasma levels are also defined by specific genotypes, with ε2/ε2 participants having the highest ApoE levels and ε4/ε4 participants having the lowest (Figure 3). A decrease in the amount of available plasma ApoE among ε4 allele carriers could have significant implications in the disease process. Given the involvement of ApoE in Aβ clearance and lipid transport, this seems mechanistically plausible. *APOE*-ε4 carriers have been shown to have increased amyloid burden, and this may be due to decreased clearance from the brain resulting from the limited ApoE available to bind Aβ [17,19]. Looking more closely at the literature regarding brain ApoE levels, this concept has also been demonstrated in targeted replacement mice, in which genotype clearly affected ApoE levels specifically in the CNS (with ε4/ε4 mice having the least brain ApoE [34]). Also, reduced ApoE plasma and CNS levels correlated with the development of AD, suggesting a direct consequence of having less ApoE. The ApoE4 isoform is reportedly less stable and may be preferentially degraded compared with ApoE3 in astrocytes, providing a possible biological explanation for the decrease in protein availability in this particular genotype [34].

ApoE isoforms are known to differentially transport and regulate cholesterol levels because of their amino acid differences, with ApoE4 preferentially binding to low-density lipoprotein and ApoE2 or ApoE3 binding to high-density lipoprotein [34]. Cholesterol uptake is also in part dependent upon the ApoE isoform bound to the lipid because ApoE4-mediated cholesterol uptake has been shown to be lower [35,36]. It is likely, therefore, that peripheral ApoE levels, as determined by individual isoforms, have a direct effect on lipid transport and cholesterol levels. *APOE*-ε4 carriers, having insufficient ApoE, may have reduced distribution of cholesterol to neurons for important functions such as membrane maintenance, repair and synaptogenesis, which are crucial for learning and memory [34,37,38]. With AD subjects exhibiting lower ApoE levels in this cohort, this may have similar implications because over 68% of the AIBL AD group carries the *APOE*-ε4 allele as well. Interestingly, researchers have used animal models to demonstrate that different ApoE isoforms also predict varying outcomes in response to CNS injury [13]. In this regard, ApoE deficient mice had a complete inability to recuperate from experimentally induced head injury, illustrating the essential role of this protein in neuronal repair [39].

Also of potential significance in considering the downstream effects of ApoE levels is that the cholinergic pathway is highly dependent upon lipid homeostasis for the synthesis of acetylcholine [40]. Cholinergic dysfunction is a well-documented feature of AD, where many treatment strategies have revolved around augmenting levels of this particular neurotransmitter [12,41]. The link between these two systems is lipid maintenance, which illustrates the potential importance of ApoE in this pathway. Because the AD group in this study exhibited significantly lower levels of this protein, and considering the subsequent biological implications described here, plasma ApoE may be an important element in a predictive biomarker panel for early diagnosis.

The *APOE*-ε4 allele not only is considered a risk factor for AD, but is now also being used as a predictor for cognitive decline. Cognitively normal *APOE*-ε4 allele carriers have been shown to exhibit an increase in amyloid burden as measured by PiB-PET [42]. Additionally, those who are cognitively healthy *APOE*-ε4 carriers have exhibited structural damage and associated cognitive decline compared with non-E4 carriers as observed by magnetic resonance imaging (MRI) [43,44]. With a clear reduction in ApoE protein levels associated with the carriage of the *APOE*-ε4 allele, and with the utilisation of MRI and PET neuroimaging, we are a step closer to understanding the consequences of reduced ApoE levels. In terms of the relationship between ApoE protein levels and amyloid burden in the present study, a subset of participants who underwent PiB-PET imaging were analysed separately with respect to plasma ApoE levels. A significant negative correlation was found between SUVR and ApoE levels, suggesting that lower circulating ApoE levels are associated with higher amyloid burden in the brain. Again, this supports our previous work, which demonstrated a similar significant result [22]. Given the relationship between ApoE isoforms and subsequent protein levels, ApoE could perhaps play an interchangeable role as a risk factor and/or biomarker.

To strengthen our findings, we used ApoE levels at the 18-month follow-up to predict neocortical Aβ burden based on PiB-PET-determined SUVR. An AUC of 60% was observed for neocortical Aβ burden predicted with ApoE protein levels alone; however, the addition of demographic and *APOE* genotype information to the model yielded an AUC of 80% (3% above that of the demographic and *APOE* genotype information alone). The modest improvement in predicting neocortical Aβ burden with the addition of plasma ApoE levels to the base model demonstrated a possible application for plasma ApoE levels in a clinical setting and its importance to the increased accuracy for potential population screening protocols to identify individuals at increased risk of developing AD.

## Conclusions

The findings reported here are from the 18-month follow-up time point of the longitudinal AIBL study. The mean plasma ApoE levels are lower in the MCI and AD clinical categories than in the HC participants in the age, sex and *APOE*-ε4 genotype controlled data set. The differences in mean ApoE levels observed among the clinical categories are consistent with the previously published baseline results from AIBL and also reiterate that *APOE*-ε4 carriers have the lowest levels of plasma ApoE levels. This study gives the insight that lower levels of ApoE could have major implications in contributing to the progression of AD as also observed by its negative correlation with neocortical amyloid burden as measured by PiB-PET. On the basis of these consistent results derived from a large, well-characterised cohort, ApoE has the potential to become an important biomarker target for the early diagnosis of AD.

## Abbreviations

Aβ: Amyloid-β
AD: Alzheimer’s disease
AIBL: Australian Imaging, Biomarkers and Lifestyle Study of Ageing
ANOVA: Analysis of variance
APOE: Apolipoprotein E
AUC: Area under the curve
CI: Confidence interval
CNS: Central nervous system
^11^C-PiB-PET: Carbon-11-labeled Pittsburgh Compound B positron emission tomography
HC: Healthy control
HSD: Honestly significant difference
MCI: Mild cognitive impairment
MRI: Magnetic resonance imaging
PET: Positron emission tomography
PiB: Pittsburgh Compound B
SD: Standard deviation
SUV: Standardised uptake value
SUVR: Standardised uptake value ratio

## References

[1] Tarawneh R, Holtzman DM (2010). Biomarkers in translational research of Alzheimer’s disease. Neuropharmacology..

[2] Thambisetty M, Lovestone S (2010). Blood-based biomarkers of Alzheimer’s disease: challenging but feasible. Biomark Med..

[3] Rowe CC, Ellis KA, Rimajova M, Bourgeat P, Pike KE, Jones G (2010). Amyloid imaging results from the Australian Imaging, Biomarkers and Lifestyle (AIBL) study of aging. Neurobiol Aging..

[4] Rowe CC, Ng S, Ackermann U, Gong SJ, Pike K, Savage G (2007). Imaging β-amyloid burden in aging and dementia. Neurology..

[5] Villemagne VL, Burnham S, Bourgeat P, Brown B, Ellis KA, Salvado O (2013). Amyloid β deposition, neurodegeneration, and cognitive decline in sporadic Alzheimer’s disease: a prospective cohort study. Lancet Neurol..

[6] Saunders AM, Strittmatter WJ, Schmechel D, St George-Hyslop PH, Pericak-Vance MA, Joo SH (1993). Association of apolipoprotein E allele ε4 with late-onset familial and sporadic Alzheimer’s disease. Neurology..

[7] Polvikoski T, Sulkava R, Haltia M, Kainulainen K, Vuorio A, Verkkoniemi A (1995). Apolipoprotein E, dementia, and cortical deposition of β-amyloid protein. N Engl J Med..

[8] Corder EH, Saunders AM, Strittmatter WJ, Schmechel DE, Gaskell PC, Small GW (1993). Gene dose of apolipoprotein E type 4 allele and the risk of Alzheimer’s disease in late onset families. Science..

[9] Rebeck GW, Reiter JS, Strickland DK, Hyman BT (1993). Apolipoprotein E in sporadic Alzheimer’s disease: allelic variation and receptor interactions. Neuron..

[10] Mahley RW (1988). Apolipoprotein E: cholesterol transport protein with expanding role in cell biology. Science..

[11] Boyles JK, Zoellner CD, Anderson LJ, Kosik LM, Pitas RE, Weisgraber KH (1989). A role for apolipoprotein E, apolipoprotein A-I, and low density lipoprotein receptors in cholesterol transport during regeneration and remyelination of the rat sciatic nerve. J Clin Invest..

[12] Poirier J (2000). Apolipoprotein E, and Alzheimer’s disease: a role in amyloid catabolism. Ann N Y Acad Sci..

[13] Liu CC, Kanekiyo T, Xu H, Bu G (2013). Apolipoprotein E and Alzheimer disease: risk, mechanisms and therapy. Nat Rev Neurol..

[14] LaDu MJ, Falduto MT, Manelli AM, Reardon CA, Getz GS, Frail DE (1994). Isoform-specific binding of apolipoprotein E to β-amyloid. J Biol Chem..

[15] Yang DS, Smith JD, Zhou Z, Gandy SE, Martins RN (1997). Characterization of the binding of amyloid-β peptide to cell culture-derived native apolipoprotein E2, E3, and E4 isoforms and to isoforms from human plasma. J Neurochem..

[16] Strittmatter WJ, Saunders AM, Schmechel D, Pericak-Vance M, Enghild J, Salvesen GS (1993). Apolipoprotein E: high-avidity binding to β-amyloid and increased frequency of type 4 allele in late-onset familial Alzheimer disease. Proc Natl Acad Sci U S A..

[17] Strittmatter WJ, Weisgraber KH, Huang DY, Dong LM, Salvesen GS, Pericak-Vance M (1993). Binding of human apolipoprotein E to synthetic amyloid β peptide: isoform-specific effects and implications for late-onset Alzheimer disease. Proc Natl Acad Sci U S A..

[18] Evans KC, Berger EP, Cho CG, Weisgraber KH, Lansbury PT (1995). Apolipoprotein E is a kinetic but not a thermodynamic inhibitor of amyloid formation: implications for the pathogenesis and treatment of Alzheimer disease. Proc Natl Acad Sci U S A..

[19] Schmechel DE, Saunders AM, Strittmatter WJ, Crain BJ, Hulette CM, Joo SH (1993). Increased amyloid β-peptide deposition in cerebral cortex as a consequence of apolipoprotein E genotype in late-onset Alzheimer disease. Proc Natl Acad Sci U S A..

[20] Vemuri P, Wiste HJ, Weigand SD, Knopman DS, Shaw LM, Trojanowski JQ (2010). Effect of apolipoprotein E on biomarkers of amyloid load and neuronal pathology in Alzheimer disease. Ann Neurol..

[21] Berlau DJ, Corrada MM, Head E, Kawas CH (2009). APOE ε2 is associated with intact cognition but increased Alzheimer pathology in the oldest old. Neurology..

[22] Gupta VB, Laws SM, Villemagne VL, Ames D, Bush AI, Ellis KA (2011). Plasma apolipoprotein E and Alzheimer disease risk: the AIBL study of aging. Neurology..

[23] Ellis KA, Bush AI, Darby D, De Fazio D, Foster J, Hudson P (2009). The Australian Imaging, Biomarkers and Lifestyle (AIBL) study of aging: methodology and baseline characteristics of 1112 individuals recruited for a longitudinal study of Alzheimer’s disease. Int Psychogeriatr..

[24] Tierney MC, Fisher RH, Lewis AJ, Zorzitto ML, Snow WG, Reid DW (1988). The NINCDS-ADRDA Work Group criteria for the clinical diagnosis of probable Alzheimer’s disease: a clinicopathologic study of 57 cases. Neurology..

[25] Hixson JE, Vernier DT (1990). Restriction isotyping of human apolipoprotein E by gene amplification and cleavage with *Hha*I. J Lipid Res..

[26] Pike KE, Savage G, Villemagne VL, Ng S, Moss SA, Maruff P (2007). β-amyloid imaging and memory in non-demented individuals: evidence for preclinical Alzheimer’s disease. Brain.

[27] Burnham SC, Faux NG, Wilson W, Laws SM, Ames D, Bedo J (2014). A blood-based predictor for neocortical Aβ burden in Alzheimer’s disease: results from the AIBL study. Mol Psychiatry..

[28] R: A language and environment for statistical computing. http://www.R-project.org/. Accessed 14 Mar 2015.

[29] Siest G, Bertrand P, Qin B, Herbeth B, Serot JM, Masana L (2000). Apolipoprotein E polymorphism and serum concentration in Alzheimer's disease in nine European centres: the ApoEurope study. Clin Chem Lab Med..

[30] Wang C, Yu JT, Wang HF, Jiang T, Tan CC, Meng XF (2014). Meta-analysis of peripheral blood apolipoprotein E levels in Alzheimer’s disease. PLoS One..

[31] Cruchaga C, Kauwe JS, Nowotny P, Bales K, Pickering EH, Mayo K (2012). Cerebrospinal fluid APOE levels: an endophenotype for genetic studies for Alzheimer’s disease. Hum Mol Genet..

[32] Altmann A, Tian L, Henderson VW, Greicius MD (2014). Alzheimer’s Disease Neuroimaging Initiative Investigators. Sex modifies the APOE-related risk of developing Alzheimer disease. Ann Neurol..

[33] Raffai RL, Dong LM, Farese RV, Weisgraber KH (2001). Introduction of human apolipoprotein E4 “domain interaction” into mouse apolipoprotein E. Proc Natl Acad Sci U S A..

[34] Riddell DR, Zhou H, Atchison K, Warwick HK, Atkinson PJ, Jefferson J (2008). Impact of apolipoprotein E (ApoE) polymorphism on brain ApoE levels. J Neurosci..

[35] Rapp A, Gmeiner B, Hüttinger M (2006). Implication of apoE isoforms in cholesterol metabolism by primary rat hippocampal neurons and astrocytes. Biochimie..

[36] Leoni V (2011). The effect of apolipoprotein E (ApoE) genotype on biomarkers of amyloidogenesis, tau pathology and neurodegeneration in Alzheimer’s disease. Clin Chem Lab Med..

[37] Pfrieger FW (2003). Role of cholesterol in synapse formation and function. Biochim Biophys Acta..

[38] Mauch DH, Nägler K, Schumacher S, Göritz C, Müller EC, Otto A (2001). CNS synaptogenesis promoted by glia-derived cholesterol. Science..

[39] Chen Y, Lomnitski L, Michaelson DM, Shohami E (1997). Motor and cognitive deficits in apolipoprotein E-deficient mice after closed head injury. Neuroscience..

[40] Blusztajn JK, Liscovitch M, Richardson UI (1987). Synthesis of acetylcholine from choline derived from phosphatidylcholine in a human neuronal cell line. Proc Natl Acad Sci U S A..

[41] Perry EK (1980). The cholinergic system in old age and Alzheimer’s disease. Age Ageing..

[42] Pike KE, Ellis KA, Villemagne VL, Good N, Chetelat G, Ames D (2011). Cognition and β-amyloid in preclinical Alzheimer’s disease: data from the AIBL study. Neuropsychologia..

[43] Espeseth T, Westlye LT, Fjell AM, Walhovd KB, Rootwelt H, Reinvang I (2008). Accelerated age-related cortical thinning in healthy carriers of apolipoprotein E ε4. Neurobiol Aging..

[44] Fennema-Notestine C, Panizzon MS, Thompson WR, Chen CH, Eyler LT, Fischl B (2011). Presence of ApoE ε4 allele associated with thinner frontal cortex in middle age. J Alzheimers Dis..

